# Higher Education

**Published:** 1897-08

**Authors:** J. B. Ramsey

**Affiliations:** Emmet, Texas


### Higher Education.

EMMET, TEXAS, June 30, 1897.

*Editors Texas Medical Journal:*

While we complain at our legislators for their failure to enact a law that will regulate the practice of medicine in Texas, I believe the greatest trouble lies with ourselves. It seems that many of the laity delight to see doctors disagreeing and pulling against each other. This state of affairs should not exist. Instead of a physician regarding his fellow-practitioner as an enemy, as is often the case, he should look upon him as a friend bound by ties that cannot be ignored, or should not be. I have given this subject considerable study, and my conclusions are not drawn from a few instances, nor confined to one locality; but I find it the case everywhere.

The medical ethics are almost disregarded by many. I have known doctors to solicit practice by agreeing to practice cheaper than other physicians; some of these claiming to be graduates of regular schools of medicine. It is common for the consulting physician to give his opinion about the case he has been called to see to everybody he sees, and will even call, in the absence of the attending physician, and make suggestions in regard to treatment, etc. We can never hope to command the respect of the public so long as these things exist. I don't believe the medical profession is looked upon now with as high regard as in former days, because we do not respect each other sufficiently. We should be united in one common brotherhood and pull together to uphold the dignity of the profession. It has come to the point where an honorable and ethical physician can barely make a living, and it is not because there are so many doctors, but because of so much strife and contention among us. This should not be; rather let us charge a reasonable fee for our professional services and turn our backs upon all but those who are duly qualified to practice, and urge our medical examiners to rigidly enforce the existing law until we can get a better one passed by our legislature. Let us wake up to a realization of our duty to each other and to the people, and the profession of medicine in Texas will be second to none. Let us not cease our efforts to raise the standard of medical education by prevailing upon legislators to pass a law that will cause every man who proposes to practice in this State to stand a thorough examination by a competent State board of examiners, diploma or no diploma. "United we stand, divided we fall."

J. B. RAMSEY, M. D.

